# Impact of Elmiron adjunct therapy on outcomes of fulguration in chronic interstitial cystitis in women

**DOI:** 10.1186/s12894-026-02057-w

**Published:** 2026-01-24

**Authors:** Sezgin Yeni, Mete Kilciler

**Affiliations:** 1 Mudanya University, Vocational School, Bursa, Turkey; 2 Vm Medical Park Bursa Hospital, Bursa, Turkey

**Keywords:** Interstitial cystitis, Hunner’s lesions, Pentosan polysulfate sodium, Elmiron, Fulguration, Chronic pelvic pain

## Abstract

**Background:**

Interstitial cystitis (IC), particularly in patients with Hunner-type lesions, is a chronic, debilitating condition with limited treatment success. While bladder fulguration and pentosan polysulfate sodium (PPS, Elmiron) are established treatments, their combined efficacy has not been systematically studied.

**Objective:**

To evaluate whether adjunctive Elmiron therapy enhances the therapeutic outcomes of fulguration in female patients with Hunner-type IC.

**Methods:**

A total of 97 female patients with Hunner-type IC who underwent bladder fulguration between 2022 and 2024 were included in this retrospective analysis of prospectively collected data. Group 1 (*n* = 49) received Elmiron therapy for 6 months postoperatively, while Group 2 (*n* = 48) did not. Patients were followed for 12 months, and outcomes were assessed using the Visual Analogue Scale (VAS), Interstitial Cystitis Symptom Index (ICSI), and Interstitial Cystitis Problem Index (ICPI).

**Results:**

No statistically significant differences were observed between groups in VAS scores at any time point. However, Group 1 showed a significantly greater reduction in ICSI scores at 12 months (*p* = 0.023) and in ICPI scores at both 6 months (*p* = 0.040) and 12 months (*p* < 0.001). Percentage change analyses confirmed a more pronounced improvement in symptom and problem indices in the Elmiron group at 12 months.

**Conclusion:**

Adjunctive Elmiron therapy appears to enhance the long-term symptom and problem index outcomes of fulguration in women with Hunner-type interstitial cystitis. Further large-scale, multicenter studies are warranted to optimize treatment protocols and establish standardized guidelines for this combination approach.

**Supplementary Information:**

The online version contains supplementary material available at 10.1186/s12894-026-02057-w.

## Introduction

Interstitial Cystitis/Painful Bladder Syndrome (IC/PBS) is a chronic condition of unknown etiology that affects lower urinary tract function, causes pelvic pain, and significantly disrupts psychological well-being and sexual health. It is more prevalent in women, with an estimated incidence of 100–300 per 100,000 women. In men, the prevalence is approximately 10–20% of that observed in women [1].

Although various treatment options exist for IC, there is currently no therapy that can fully relieve symptoms or cure the disease. Treatment modalities for IC include bladder fulguration, bladder hydrodistention, oral medications such as amitriptyline, intravesical therapies like dimethyl sulfoxide (DMSO) and pentosan polysulfate sodium (PPS) [2, 3].

Hunner’s ulcers are present in approximately 20% of patients with interstitial cystitis. Patients with Hunner lesions often respond poorly to standard treatments, and their symptoms may be so severe that cystectomy becomes the only effective option for relief [4].

Elmiron therapy and the electrosurgical management of Hunner’s ulcers are among the recognized treatment strategies for IC [5, 6]. The aim of our study was to determine whether combining these two treatment modalities in patients with Hunner-type IC enhances therapeutic efficacy.

## Materials and methods

This study was approved by the Mudanya University Health Sciences Ethics Committee (Ref: E-40839601-50.04-63) and conducted in accordance with the Declaration of Helsinki (1975, revised 2008). Patient data were collected prospectively and analyzed retrospectively. Written informed consent was obtained from all participants.

### Patient selection and diagnostic criteria

The diagnosis of interstitial cystitis/bladder pain syndrome (IC/BPS) was established based on clinical symptoms and the exclusion of other causes of lower urinary tract symptoms, in accordance with the European Society for the Study of Interstitial Cystitis (ESSIC) criteria [7]. Cystoscopy was not used as a primary diagnostic tool, but was performed to exclude other bladder pathologies, to identify Hunner lesions, and as part of therapeutic hydrodistension.

Between 2022 and 2024, a total of 352 female patients underwent cystoscopy at Mudanya Hospital due to persistent lower urinary tract symptoms. Among these, 173 patients underwent cystoscopic hydrodistension and fulguration. After applying the exclusion criteria, 97 patients were included in the final analysis. Forty-nine patients received postoperative pentosan polysulfate sodium (Elmiron) therapy (Group 1), while 48 patients did not receive Elmiron therapy (Group 2).

All patients included in this study were consecutive female patients who underwent bladder fulguration for Hunner-type interstitial cystitis during the study period. Although patient data were collected prospectively, the analysis was performed retrospectively.

### Inclusion criteria

Female patients were included if they met all of the following criteria:

- Persistent lower urinary tract symptoms consistent with IC/BPS
- More than four episodes of cystitis per year
- Sterile urine cultures
- History of repeated antibiotic and anticholinergic use
- Visual Analog Scale (VAS) pain score > 5/10
- Interstitial Cystitis Symptom Index (ICSI) score > 10/20
- Interstitial Cystitis Problem Index (ICPI) score > 8/16

### Exclusion criteria

Patients were excluded if they had:

- Preoperative uroflowmetry findings suggestive of urethral stricture
- Active urinary tract infection (confirmed by preoperative urinalysis and urine culture)
- Chronic autoimmune diseases (e.g., Crohn’s disease, ulcerative colitis)
- Fibromyalgia
- History of gynecological or urological malignancy
- Prior chemotherapy or radiotherapy
- Neurogenic bladder requiring catheterization
- Neurological disorders (e.g., stroke, Parkinson’s disease, dementia)
- Incomplete follow-up data

### Surgical procedure

Cystoscopy was performed under anesthesia using a 26 F Olympus bipolar resectoscope with a Storz camera system. The bladder was hydrodistended at a pressure of 80 cm H₂O for 3 min. Fulguration was performed exclusively on lesions consistent with Hunner-type inflammatory lesions, in accordance with the institutional protocol. Non-specific petechial hemorrhages (glomerulations) were not considered treatment targets and were not routinely fulgurated. No routine bladder biopsies were obtained.

An 18 F Foley catheter was placed postoperatively and removed after 4 h. All patients were discharged on the same day.

### Study design and follow-up

This study was not randomized. Following bladder fulguration, patients were allocated to the Elmiron or non-Elmiron group based on patient preference and treatment tolerance.

At the 2-week postoperative follow-up, patients were offered oral pentosan polysulfate sodium (Elmiron) therapy for 6 months as adjunctive treatment. Patients who accepted and completed the treatment were assigned to Group 1, while those who declined therapy or discontinued Elmiron due to adverse effects (e.g., nausea, dizziness, fatigue) were assigned to Group 2.

Adherence to Elmiron therapy was assessed through prescription records and patient self-report obtained during routine postoperative follow-up visits.

Demographic and clinical variables collected included age, body mass index (BMI), smoking status, menopausal status, diabetes mellitus, hypertension, allergy history, and symptom duration. Symptom severity was assessed using validated patient-reported outcome measures, including the Visual Analog Scale (VAS), Interstitial Cystitis Symptom Index (ICSI), and Interstitial Cystitis Problem Index (ICPI). No additional subjective, functional, or objective parameters—such as frequency–volume charts, urodynamic studies, or follow-up cystoscopic findings—were routinely recorded.

Patients were followed for 12 months to assess mid-term outcomes, and outcomes were compared between the two groups.

### Statistical analysis

An a priori power analysis was performed to determine the required sample size, referencing the study by Chuang et al. (2020) [8]. Since the reference study reported means and 95% confidence intervals (CI) rather than standard deviations, the standard deviations were estimated using the formula SD = $\:\sqrt{n}$ x (Upper Limit - Lower Limit) / 3.92. The divisor 3.92 corresponds to the standardized width of the 95% confidence interval (derived from 2 × 1.96). Based on this calculation, the baseline Visual Analog Scale (VAS) pain scores were determined as 4.6 ± 2.4 for the Placebo group and 6.2 ± 2.4 for the Elmiron group. The effect size calculated from these data was found to be Cohen’s d = 0.67. Assuming a Type I error rate of 5% (α = 0.05) and a power of 85%, the analysis indicated that a minimum of *n* = 41 patients per group, and a total of *n* = 82 patients, were required. To account for a potential 15% sample attrition, the target sample size was revised to n_t_ = 96 using the n / (1 − 0.15) formula, resulting in a decision to include a total of patients in the study. All analyses were conducted using G*Power 3.1.9.7 software.

The normality of continuous variables was assessed using the Shapiro–Wilk test. Normally distributed variables are presented as mean ± standard deviation, while non-normally distributed variables are presented as median (minimum–maximum). Categorical variables are expressed as counts and percentages (n [%]).

Comparisons between the two groups were performed using the Independent Samples t-test for normally distributed variables and the Mann–Whitney U test for non-normally distributed variables. Preoperative and postoperative values were compared using the Wilcoxon signed-rank test. Categorical variables were analyzed using the Pearson chi-square test.

All statistical analyses were performed using SPSS version 25.0 (IBM Corp., Armonk, NY, USA). A p-value < 0.05 was considered statistically significant.

## Results

A detailed examination of Table 1 reveals no statistically significant differences between the groups receiving and not receiving Elmiron treatment postoperatively with respect to age distribution (*p* = 0.395). Similarly, the groups did not differ significantly in terms of body mass index (BMI) distribution (*p* = 0.491).

**Table 1:** Comparison of demographic and clinical characteristics between groups

	Group 1 (n=49)	Group 2 (n=48)	p value
Age (year)	56.5 ± 11.9	54.4 ± 10.7	0.395
BMI (kg/m2)	28.1 (20.8-38.7)	27.9 (18.6-41.7)	0.491
Smoking			0.773
• Yes	14 (28.6%)	15 (31.3%)	
• No	35 (71.4%)	33 (68.8%)	
Menopause	31 (63.3%)	36 (75.0%)	0.211
DM	13 (26.5%)	11 (22.9%)	0.680
HT	25 (51.0%)	22 (45.8%)	0.609
Allergy	15 (30.6%)	10 (20.8%)	0.271
Symptom duration (year)	3 (1-12)	3 (1-10)	0.668

Data are expressed as mean ± standard deviation, median (minimum–maximum), and number (%)

*BMI* Body Mass Index, *DM* Diabetes Mellitus, *HT* Hypertension

No statistically significant differences were also observed between the groups regarding smoking status (*p* = 0.773) and menopausal status (*p* = 0.211). Moreover, the prevalence of diabetes mellitus (DM), hypertension (HT), allergies, and symptom duration did not differ significantly between the groups (all p-values > 0.05) (Table 1).

No significant difference was observed between the study groups with respect to preoperative Visual Analog Scale (VAS) scores (*p* = 0.938). Likewise, no statistically significant differences were detected between the Elmiron and non-Elmiron groups at the 1st, 3rd, 6th, and 12th postoperative months based on VAS score assessments (*p* > 0.05 at all time points) (Table 2).

**Table 2:** Comparison of VAS, ICSI, and ICPI scores between groups

		Group 1 (n=49)	Group 2 (n=48)	p value
VAS Score (0-10)				
Preoperative		10(8-10)	10(8-10)	0.938
1. Month		2(0-9)	2.5(0-9)	0.924
3. Month		1(0-6)	1(0-8)	0.878
6. Month		1(0-7)	0.5(0-9)	0.812
12. Month		1(0-7)	1(0-9)	0.317
ICSI Score (0-20)				
Preoperative		18(15-20)	18(12-20)	0.610
1. Month		7(0-16)	7(0-17)	0.597
3. Month		7(0-10)	5(0-16)	0.644
6. Month		7(0-10)	7(0-16)	0.450
12. Month		9(0-12)	9(0-15)	**0.023**
		6.8±4.2	8.3±4.4	
ICPI Score (0-16)				
Preoperative		14(10-16)	13.5(8-16)	0.734
1. Month		5(0-9)	6(0-10)	0.081
3. Month		3(0-9)	5(0-10)	0.082
6. Month		5(0-9)	7(0-10)	**0.040**
12. Month		7(0-9)	9(0-10)	**<0.001**

Bold values indicate statistically significant differences between groups (*p* < 0.05)

*VAS* Visual Analogue Scale

*ICSI* Interstitial Cystitis Symptom Index

*ICPI* Interstitial Cystitis Problem Index

Regarding preoperative symptom index scores, no significant difference was found between the groups (*p* = 0.610). Postoperative evaluations at the 1st, 3rd, and 6th months similarly revealed no significant differences in symptom index scores between the Elmiron and non-Elmiron groups (*p* > 0.05). However, at the 12th postoperative month, the Elmiron group demonstrated a significantly lower mean symptom index score (6.8 ± 4.2) compared to the non-Elmiron group (8.3 ± 4.4) (*p* = 0.023).

No statistically significant difference was noted in preoperative problem index scores between the groups (*p* = 0.734). At the 1st and 3rd postoperative months, problem index scores remained comparable (*p* > 0.05). Nevertheless, at the 6th month, the median problem index score was significantly lower in the Elmiron group [5 (range: 0–9)] compared to the non-Elmiron group [7 (range: 0–10)] (*p* = 0.040). This trend persisted at the 12th month, with the Elmiron group exhibiting a median problem index of 7 (range: 0–9) versus 9 (range: 0–10) in the non-Elmiron group, reflecting a statistically significant difference (*p* < 0.001).

**Fig. 1:**
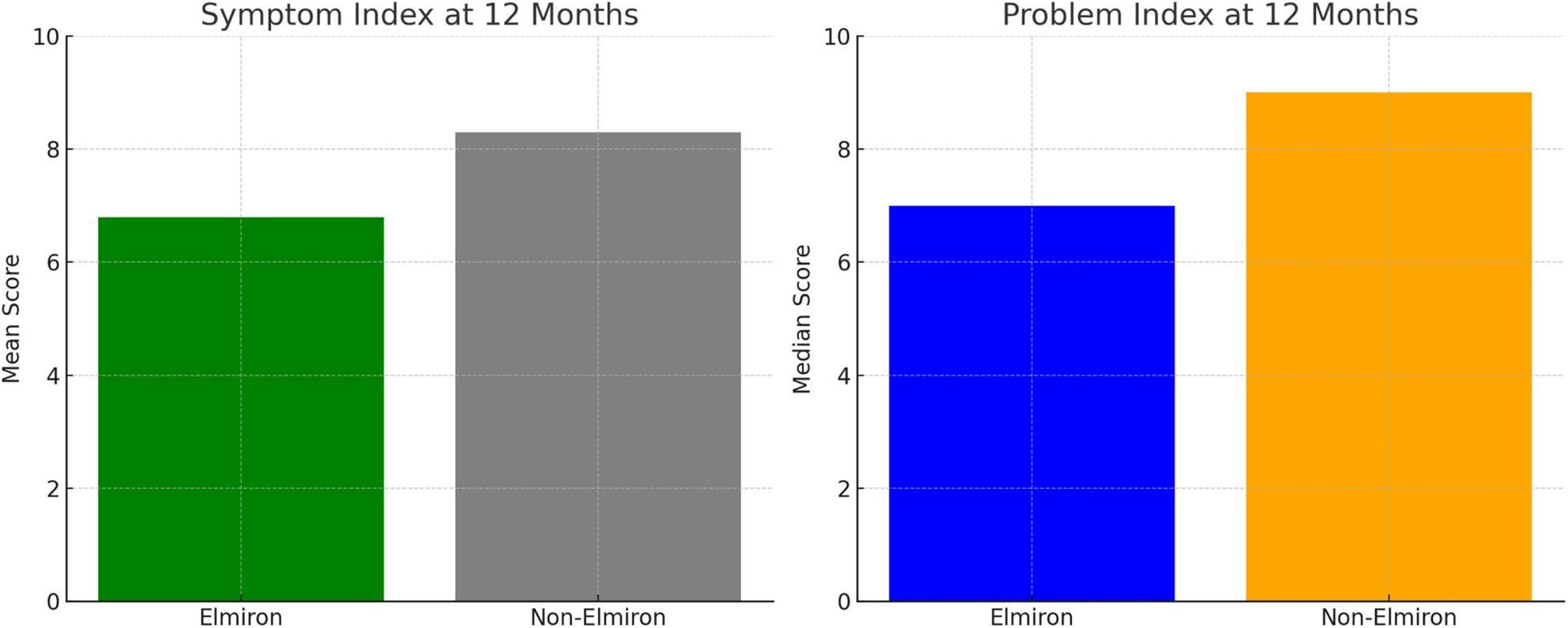
Comparison of symptom index and problem index between groups at 12 months

At the 12th month, the mean Symptom Index and Problem Index was significantly lower in the Elmiron group (Fig. 1). These charts visually support the finding that Elmiron group led to greater improvement in both symptom and problem indices, particularly at the 12-month follow-up (Fig. 1).

## Discussion

In this study, we employed a combination of fulguration therapy and PPS treatment, both of which have previously demonstrated efficacy in the management of patients with interstitial cystitis. Our primary aim was to investigate whether combining these two therapeutic modalities would produce a potentiated therapeutic outcome. Previous studies suggest that while one of these treatments targets the underlying lesion, the other acts by reducing the permeability of the urothelial mucosa, thereby offering symptomatic relief [9]. To our knowledge, there is no prior study in the literature that has evaluated this specific combination therapy.

Fulguration treatment induces scar tissue formation in the bladder mucosa. This scar tissue, unlike normal mucosa, has reduced permeability. Consequently, it limits the transurothelial passage of harmful substances and irritants present in the urine [10]. Elmiron therapy functions similarly by reinforcing the glycosaminoglycan (GAG) layer of the bladder [11]. Therefore, in patients with IC, the combination of these two treatments may further reduce bladder mucosal permeability, thereby potentiating therapeutic efficacy. This hypothesis served as the rationale for conducting the present study.

In our study, the mean symptom index score at 12 months was significantly lower in Group 1 (Elmiron-treated group) compared to Group 2, indicating a statistically greater reduction in symptoms. Similarly, the mean problem index scores at both the 6th and 12th postoperative months were significantly lower in Group 1. Importantly, these statistically significant improvements were also clinically meaningful: when evaluated against the minimal clinically important difference (MCID) thresholds defined by Propert et al., the magnitude of reduction observed in both ICSI and ICPI scores in the Elmiron group exceeded the level associated with a “markedly improved” patient-reported outcome [12]. This finding suggests that the benefit of adjunctive Elmiron therapy extends beyond statistical significance and translates into a substantial, clinically relevant improvement in patient symptoms.

We believe the absence of a significant change in VAS scores may be attributed to the fact that fulguration was performed only once in our study. Fulguration therapy helps by reducing local sources of inflammation and irritation of nerve endings. In the long term, it promotes scar tissue formation in the bladder mucosa, which reduces its permeability [10]. However, a single application of fulguration was not expected to result in a statistically significant change in VAS scores.

Previous studies have shown that fulguration therapy, when performed periodically, does not reduce bladder capacity [13]. Therefore, we opted not to perform hydrodistension in our follow-up assessments, as we did not anticipate a decrease in bladder capacity due to our combination therapy in IC patients.

Recent reports have described progressive maculopaty associated with PPS administration, resulting in visual impairment [14]. Accordignly, PPS was employed at the lowest effective therapeutic dose and was not administered beyond the durations specified in our study. As a result, no vision-related complications were observed in any of the patients.

Our study indicates that the combination of fulguration and Elmiron therapy may represent an effective approach in reducing symptoms in patients with interstitial cystitis. However, there remain unanswered questions regarding whether fulguration should be repeated during follow-up, and if so, at what intervals. Similarly, it is still unclear whether Elmiron should be administered orally or intravesically, the optimal timing of administration following fulguration, appropriate dosing, and the ideal treatment intervals.

### Limitation

The retrospective analysis of prospectively collected data represents an inherent limitation of this study. In addition, treatment allocation was not randomized, as patients self-selected into the Elmiron or non-Elmiron groups based on preference or treatment tolerance, which may have introduced selection bias.

This was a single-center study, which may limit the generalizability of the findings. Nevertheless, an a priori power analysis demonstrated that the sample size was sufficient to detect clinically meaningful differences between the study groups.

The 12-month follow-up period was chosen to evaluate mid-term treatment outcomes. However, previous studies have reported that the therapeutic effects of bladder fulguration may persist for 6–18 months. Therefore, longer follow-up durations may be required to fully assess the durability of adjunctive Elmiron therapy and to differentiate its effects from the primary benefits of fulguration or bladder hydrodistension.

Furthermore, objective parameters such as frequency–volume charts or follow-up cystoscopic findings were not routinely recorded, which limited the ability to correlate patient-reported outcomes with objective measures of treatment response.

## Conclusion

While our findings suggest that combination therapy may offer a promising treatment strategy, we believe that larger, multicenter studies are necessary to determine the optimal therapeutic protocol and to clarify which factors influence treatment efficacy. Only through such investigations can this combination approach be validated as a standardized treatment concept for interstitial cystitis.

## Supplementary Information

Supplementary Material 1.

Supplementary Material 2.

## Data Availability

The datasets used and/or analysed during the current study are available from the corresponding author on reasonable request.

## Abbreviations

IC: Interstitial cystitis
IC/BPS: Interstitial cystitis/bladder pain syndrome
PPS: Pentosan polysulfate sodium
VAS: Visual Analogue Scale
ICSI: Interstitial Cystitis Symptom Index
ICPI: Interstitial Cystitis Problem Index
BMI: Body Mass Index
ESSIC: European Society for the Study of Interstitial Cystitis
DM: Diabetes Mellitus
HT: Hypertension
