# Supplementary material for: Impact of Elmiron adjunct therapy on outcomes of fulguration in chronic interstitial cystitis in women
Source: BMC Urol. 2026 Jan 24;26:48. doi: 10.1186/s12894-026-02057-w (PMC12911371; doi:10.1186/s12894-026-02057-w)
Supplement: Supplementary file 1 — Supplementary Material 1. [file 12894_2026_2057_MOESM1_ESM.docx]

**Table 1:** Comparison of Demographic and Clinical Characteristics Between Groups

|  | **Group 1 (n=49)** | **Group 2 (n=48)** | **p value** |
| --- | --- | --- | --- |
| **Age (year)** | 56.5 ± 11.9 | 54.4 ± 10.7 | 0.395 |
|  |  |  |  |
| **BMI (kg/m^2^)** | 28.1 (20.8-38.7) | 27.9 (18.6-41.7) | 0.491 |
|  |  |  |  |
| **Smoking** |  |  | 0.773 |
| - **Yes** | 14 (28.6%) | 15 (31.3%) |  |
| - **No** | 35 (71.4%) | 33 (68.8%) |  |
|  |  |  |  |
| **Menopause** | 31 (63.3%) | 36 (75.0%) | 0.211 |
|  |  |  |  |
| **DM** | 13 (26.5%) | 11 (22.9%) | 0.680 |
|  |  |  |  |
| **HT** | 25 (51.0%) | 22 (45.8%) | 0.609 |
|  |  |  |  |
| **Allergy** | 15 (30.6%) | 10 (20.8%) | 0.271 |
|  |  |  |  |
| **Symptom duration (year)** | 3 (1-12) | 3 (1-10) | 0.668 |

Data are expressed as mean ± standard deviation, median (minimum–maximum), and number (%).

BMI: Body Mass Index, DM: Diabetes Mellitus, HT: Hypertension

**Table 2:** Comparison of VAS, ICSI, and ICPI Scores Between Groups

|  | **Group 1 (n=49)** | | **Group 2 (n=48)** | **p value** |
| --- | --- | --- | --- | --- |
| **VAS Score (0-10)** | |  |  |  |
| Preoperative | | 10(8-10) | 10(8-10) | 0.938 |
| 1. Month | | 2(0-9) | 2.5(0-9) | 0.924 |
| 3. Month | | 1(0-6) | 1(0-8) | 0.878 |
| 6. Month | | 1(0-7) | 0.5(0-9) | 0.812 |
| 12. Month | | 1(0-7) | 1(0-9) | 0.317 |
| **ICSI Score (0-20)** | |  |  |  |
| Preoperative | | 18(15-20) | 18(12-20) | 0.610 |
| 1. Month | | 7(0-16) | 7(0-17) | 0.597 |
| 3. Month | | 7(0-10) | 5(0-16) | 0.644 |
| 6. Month | | 7(0-10) | 7(0-16) | 0.450 |
| 12. Month | | 9(0-12)  6.8±4.2 | 9(0-15)  8.3±4.4 | **0.023** |
| **ICPI Score (0-16)** | |  |  |  |
| Preoperative | | 14(10-16) | 13.5(8-16) | 0.734 |
| 1. Month | | 5(0-9) | 6(0-10) | 0.081 |
| 3. Month | | 3(0-9) | 5(0-10) | 0.082 |
| 6. Month | | 5(0-9) | 7(0-10) | **0.040** |
| 12. Month | | 7(0-9) | 9(0-10) | **<0.001** |

VAS: Visual Analogue Scale

ICSI: Interstitial Cystitis Symptom Index

ICPI: Interstitial Cystitis Problem Index
