# Supplementary figures and images for: Impact of Elmiron adjunct therapy on outcomes of fulguration in chronic interstitial cystitis in women
Source: BMC Urol. 2026 Jan 24;26:48. doi: 10.1186/s12894-026-02057-w (PMC12911371; doi:10.1186/s12894-026-02057-w)

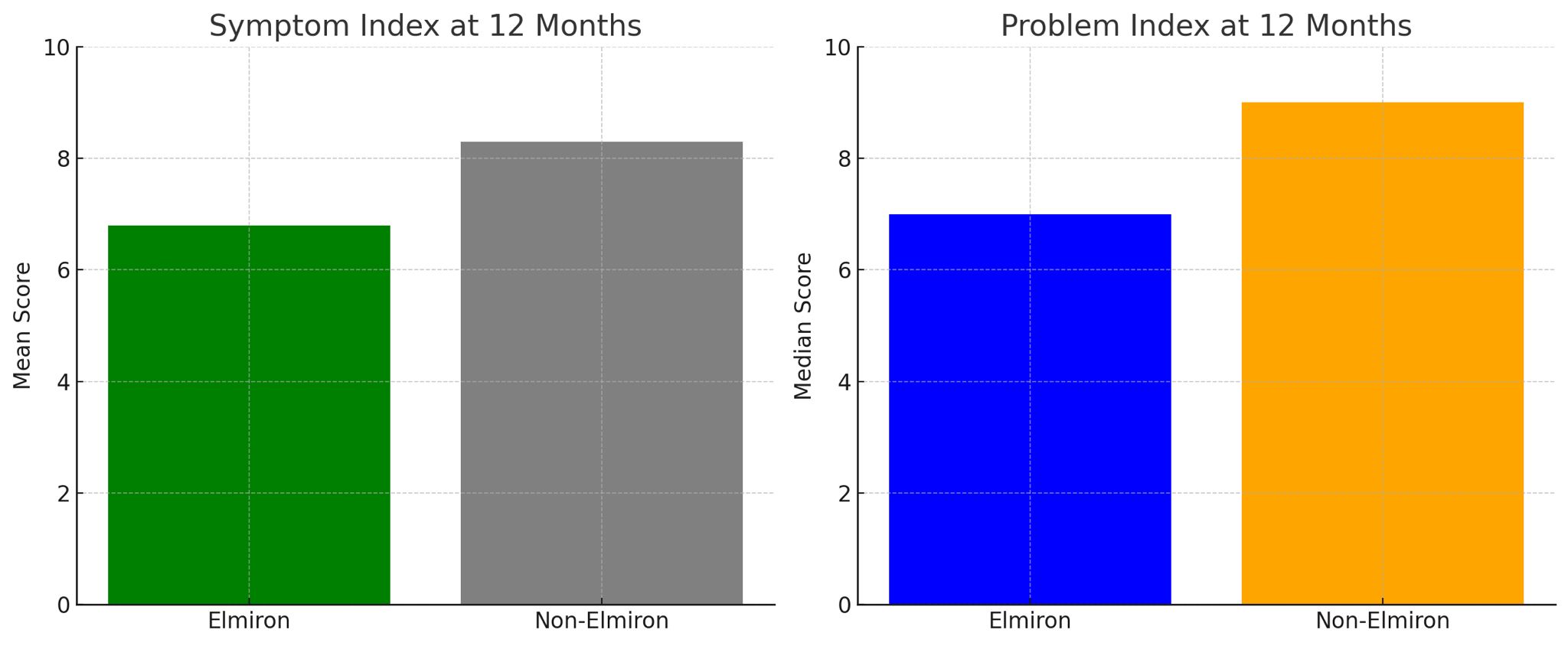

Supplement: Supplementary file 2 — Supplementary Material 2. [file 12894_2026_2057_MOESM2_ESM.docx]
